# Multi-omics integration and machine learning unveil FOXO3/SIRT1 axis as oxidative stress biomarkers and therapeutic targets in osteoporosis

**DOI:** 10.1097/MD.0000000000047356

**Published:** 2026-01-30

**Authors:** Shang Zheng, Xingye Luo, Guomin Chen, Pingxi Wang

**Affiliations:** a North Sichuan Medical College, Nanchong, Sichuan, China; b Department of Orthopedics, Dazhou Central Hospital, Dazhou, Sichuan, China.

**Keywords:** osteoporosis, oxidative stress, SIRT1/FOXO3 axis

## Abstract

Osteoporosis (OP) is a degenerative skeletal disorder marked by a decrease in bone mineral density, which significantly raises the risk of fractures. The development of OP is closely associated with oxidative stress (OS), yet the specific molecular targets of OS in the context of OP, as well as the corresponding therapeutic strategies, are not well understood. This study aimed to systematically identify OS-related biomarkers for OP and explore potential therapeutic agents, providing new insights into the pathological mechanisms and precision therapy of OP. OP-related datasets (GSE56815 as the training set and GSE35958 as the validation set) were obtained from the gene expression omnibus database. Integrated with OS-related genes from GeneCards, 72 co-expressed differentially expressed genes associated with both OP and OS were identified. A protein–protein interaction network was constructed using search tool for the retrieval of interacting genes/proteins, and 9 hub genes were identified via Maximal Clique Centrality and MCODDE algorithms. Three core genes (SIRT2, neutrophil elastase [ELANE], and forkhead box O3 [FOXO3]) were further selected using random forest and support vector machine models. Diagnostic accuracy of key genes was evaluated by receiver operating characteristic curves. Immune infiltration analysis was performed with ImmuCellAI, while transcription factors were predicted through NetworkAnalyst to establish a miRNA-seed-transcription factor (TF) regulatory network. Potential drugs were screened from DGIdb, and molecular docking was conducted with CB-DOCK2 using ligand–protein structures from PubChem and UniProt. A FOXO3/sirtuin 1 (SIRT1) functional axis was subsequently constructed. The 72 differentially expressed genes showed enrichment in cell division, chromosome segregation, and cell cycle pathways. SIRT2, ELANE, and FOXO3 exhibited high diagnostic accuracy for OP (area under the curve > 0.7). Immune infiltration analysis revealed significant differences in key immune cells and subtypes between OP and controls. The miRNA–seed–TF network implicated miR-34a and miR-132. Molecular docking confirmed stable binding of 5 compounds to targets. Critically, the FOXO3–SIRT1 axis was identified as mediating oxidative stress in OP pathogenesis. FOXO3 and SIRT2 were upregulated; ELANE downregulated. This study identified SIRT2, ELANE, and FOXO3 as key OS-related biomarkers for OP and screened 5 potential therapeutic compounds. Integrating miRNA–TF network and drug sensitivity analysis, we propose the FOXO3/SIRT1 axis as central to OP oxidative stress, providing a foundation for mechanistic studies and targeted drug development.

## 1. Introduction

Osteoporosis (OP) is a systemic skeletal disease characterized by degenerative disruption of bone microstructure and reduced bone mineral density (BMD).^[1]^ Pathologically, it is driven by a persistent imbalance between osteoblast-mediated bone formation and osteoclast-dominated bone resorption (RANKL/OPG ratio > 2.1). With the acceleration of global aging, the incidence of OP continues to rise. According to the WHO 2023 report, the prevalence of OP in Chinese women aged ≥65 years is 41.2% (95% CI: 38.5–43.8), significantly higher than the global average (35.6% for women, 9.8% for men), while the rate in Chinese men is 13.7% (95% CI: 11.2–16.1). Hip fractures associated with OP result in a 1-year mortality rate of 20%,^[2]^ posing a critical public health challenge. Recent studies have identified genetic factors (e.g., Wnt/β-catenin pathway gene mutations^[3]^), hormonal fluctuations (e.g., postmenopausal estrogen decline^[4]^), and environmental triggers (e.g., vitamin D deficiency^[5]^) as key drivers of suppressed osteoblast differentiation and hyperactive osteoclast activity. However, the molecular networks regulating this imbalance remain incompletely understood. Current clinical treatments include bisphosphonates, estrogen replacement therapy, bone-forming agents, selective estrogen receptor modulators, calcitonin, and vitamin D, which act through diverse mechanisms to improve BMD and reduce fracture risk.^[6]^ OP-related fractures are the primary cause of disability and mortality. Hip fractures, the most severe complication, lead to a 1-year mortality rate of 20% to 24% and a disability rate exceeding 50%.^[7]^ Only 40% of survivors regain independent walking ability.^[7]^ The International Osteoporosis Foundation estimates that approximately 200 million people worldwide suffer from OP, with prevalence rates of 32% in women and 12% in men over 50 years old,^[8]^ and these figures are rising due to aging populations.^[9]^ By 2040, the global OP population is projected to exceed 280 million, with Asia contributing 54% of new cases due to its large population and inadequate calcium intake.^[10]^ Oxidative stress, a pathological state caused by an imbalance between cellular oxidation and antioxidant systems, exacerbates degenerative diseases through excessive reactive oxygen species (ROS) accumulation, leading to DNA damage, protein modification, and lipid peroxidation.^[11]^ In bone metabolism, ROS bidirectionally disrupt bone mineralization by inhibiting the Runx2/Osterix pathway (Western blot analysis reveals a 60% decrease in Runx2 protein levels) and increasing osteopontin methylation (3.1-fold rise in methylation sites).^[12]^ Concurrently, experimental evidence from murine models indicates that ROS directly activate the NF-κB and MAPK pathways (phosphorylation levels ↑2.8-fold) within osteoclast precursors, thereby upregulating proteolytic enzymes like telomeric repeat amplification protocol (TRAP) and cathepsin K, which are critical for bone matrix degradation.^[13]^ Cross-sectional studies demonstrate a significant negative correlation between urinary/plasma oxidative stress markers (e.g., 8-OHdG, MDA) and lumbar spine BMD in postmenopausal OP patients (β = −0.15, *P* = .007).^[14]^ However, the lack of specific, clinically translatable oxidative stress biomarkers and incomplete elucidation of its molecular mechanisms in bone metabolism imbalance remain major challenges.^[15]^ Recent studies further highlight the pivotal role of the forkhead box O3 (FOXO3)/sirtuin 1 (SIRT1) axis in regulating oxidative stress and bone metabolic dysregulation. SIRT1 enhances FOXO3’s antioxidant activity through deacetylation (e.g., upregulating SOD2 expression), thereby mitigating ROS-induced osteoblast damage and suppressing osteoclast differentiation (50% reduction in TRAP activity).^[16]^ Clinical data reveal a positive correlation between serum SIRT1 levels and femoral neck BMD in postmenopausal OP patients (*R* = 0.29, *P* = .003).^[17]^ Nevertheless, the therapeutic potential of targeting this axis is limited by tissue specificity and NAD^+^ level fluctuations, necessitating further exploration.^[18]^

Integrating the established role of oxidative stress in OP pathogenesis and current knowledge gaps, we propose that:

Distinct oxidative stress (OS)-associated molecular regulators (including hub genes, miRNAs, and transcription factors) form dysregulated networks in osteoporosis that serve as dual-purpose diagnostic biomarkers and therapeutic targets. To validate this hypothesis, we will pursue 4 integrated objectives: identify core OP–OS mediators: integrate transcriptomic profiles (GSE56815) with OS databases using bioinformatic pipelines to screen co-associated differentially expressed genes (DEGs) and hub genes.

Quantify diagnostic potential: systematically evaluate candidate genes’ diagnostic value through ROC analysis and characterize their relationship with immune infiltration patterns.

Map regulatory architecture: reconstruct multilayer regulatory circuits (miRNA → seed gene → transcription factor [TF] networks) to explain pathological mechanisms; discover therapeutic agents: prioritize and validate drug candidates targeting key molecules via virtual screening and molecular docking simulations.

These investigations will provide mechanistic insights and preclinical evidence for developing next-generation OS-targeted OP therapeutics.

## 2. Materials and methods

### 2.1. Study overview

This retrospective bioinformatic investigation employed genomic datasets from OP patients and healthy controls (HCs), utilizing discovery cohort GSE56815 (n = 40: 20 OP/20 controls) and validation cohort GSE35958 (n = 9: 5 OP/4 controls). The analytical workflow progressed through 4 sequential phases: target screening via differential expression analysis and GeneCards integration to identify OP–OS co-expressed genes; biomarker validation using machine learning (random forest [RF]/support vector machine [SVM]) to prioritize hub genes (FOXO3/SIRT1/neutrophil elastase [ELANE]) with ROC evaluation; mechanistic exploration encompassing miRNA-TF regulatory network construction, FOXO3/SIRT1 signaling axis modeling, and immune microenvironment profiling (ImmuCellAI); and therapeutic discovery featuring molecular docking targeting FOXO3/SIRT1 axis components (CB-DOCK2). Core methodologies integrated protein interaction networks (search tool for the retrieval of interacting genes/proteins [STRING]), signaling pathway reconstruction, machine learning, and structural bioinformatics validation.

### 2.2. Data acquisition and processing

Our genomic exploration commenced with strategic data sourcing from NCBI’s gene expression omnibus (GEO) repository.^[19]^ The primary dataset analyzed was GSE56815, comprising 20 OP patients and 20 controls profiled on the GPL96 platform. Additionally, the dataset GSE35958 (5 OP/4 controls, GPL570 platform) was selected as an independent validation cohort. All datasets underwent comprehensive preprocessing and quality control using the Xiantao Platform (v2.3.1). The preprocessing workflow was iteratively optimized based on standard quality assessment metrics and included the following key steps: *background correction*: robust multi-array average background correction was employed,^[20]^ enhanced with probe-specific noise modeling to improve signal fidelity. *Normalization*: following log2 transformation, probe-level data underwent quantile normalization to standardize distributions across arrays. *Batch effect correction*: to mitigate non-biological variation, particularly between different profiling platforms, ComBat batch adjustment was applied. Manual parameter tuning (convergence tolerance = 1e‐5, maximum iterations = 100) was utilized to ensure robust correction.

Differential expression analysis was then performed on the preprocessed data using the Limma package implemented within the Xiantao Platform.^[21]^ Significance thresholds for identifying DEGs were set at an absolute log2 fold change (|log2FC|) > 0.25 and an adjusted *P*-value (adj.*P*) <.05.

Ethical approval and the need for informed consent were not applicable to this study because it involved solely the secondary analysis of preexisting, anonymized data from public databases (GEO accession numbers GSE56815 and GSE35958).

### 2.3. Screening of DEGs

Differential expression analysis was performed on the OP transcriptomic datasets using the differential expression module within the Xiantao Academic Platform. This module implements an optimized Limma algorithm to enhance statistical power in studies with limited sample sizes. The analysis was configured with automated significance thresholds set at a *P*-value <.05 and an absolute log2 fold change (|log2FC|) ≥ 0.25.

In parallel, a comprehensive list of OS-related genes was retrieved from the GeneCards database. To ensure high confidence, only entries with relevance scores ≥3 were retained,^[22]^ yielding the OS-related gene set for cross-referencing.

Potential interactions between OP-associated gene expression changes and oxidative stress pathways were investigated through Venn diagram analysis, facilitated by the platform’s “Expression Difference” module. This analysis identified the subset of genes present in both the osteoporosis differentially expressed genes (OP-DEGs) and the high-confidence OS-related gene set. The overlapping genes identified through this Venn analysis were subsequently selected for downstream functional validation and network-based analysis.

### 2.4. Functional annotation and pathway enrichment of DEGs

To characterize the functional implications of OP-associated DEGs, comprehensive functional annotation and pathway enrichment analyses were performed using the Xiantao Platform’s integrated annotation suite. *Gene ontology (GO) analysis*: we conducted GO enrichment analysis to investigate the enriched biological processes (BP),^[23]^ molecular functions, and cellular components associated with the OP-DEGs. To enhance interpretability and reduce redundancy among significant GO terms, the REVIGO algorithm was applied with a semantic similarity cutoff of 0.7. This step consolidated functionally related terms and retained nonredundant representative pathways. *Kyoto encyclopedia of genes and genomes (KEGG) pathway enrichment*: KEGG^[24]^ pathway enrichment analysis was employed to identify significant signaling pathways and regulatory networks modulated by the OP-DEGs. Only pathways meeting a significance threshold of false discovery rate adjusted *P*-value <.05 were considered statistically enriched. *Statistical thresholding*: for both GO and KEGG analyses, enriched terms and pathways with an adjusted *P*-value <.05 were selected for further consideration and downstream analysis. *Visualization*: interactive visualizations of the enrichment results were generated to facilitate interpretation. This included creating GO–KEGG bubble plots (where point color intensity represented the adjusted *P*-value) and hierarchical clustering heatmaps (using Euclidean distance as the similarity metric). These visualizations were produced utilizing the platform’s built-in ggplot2-based data visualization toolkit (version 3.4.2).^[25]^

### 2.5. Protein–protein interaction (PPI) network construction and core gene screening

PPI networks for OP-related DEGs were constructed using the STRING database (version 11.5) with a medium-confidence interaction threshold (combined score ≥ 0.7).^[26]^ The resulting networks were imported and visualized using Cytoscape software.^[27]^

To identify high-impact functional modules within the PPI network, cluster analysis was performed employing the molecular complex detection (MCODE) plugin within cytoscape, applying a clustering score threshold >2.^[28]^ Subsequently, network centrality analysis was conducted using the Maximal Clique Centrality (MCC) algorithm implemented in the CytoHubba plugin to assess node importance.^[29]^

For refinement of potential core regulatory factors, integrated machine learning-based feature selection was applied. Specifically, RF and SVM models were implemented through the Xiantao Academic Platform’s analytical modules to prioritize key genes associated with OP-oxidative stress interactions.^[30]^ Finally, comparative visualization of core gene intersections was generated using the platform’s integrated diagramming tools.

### 2.6. Diagnostic validation of core genes

To evaluate the diagnostic potential of candidate core genes identified from prior analysis, ROC analysis was implemented using Xiantao’s bioinformatics toolkit. We performed this analysis on both the primary training cohort and an independent validation dataset (GSE35958) sourced from the GEO repository.^[23]^ The platform’s ROC curve module was configured to calculate area under the curve (AUC) values with 95% confidence intervals,^[31]^ quantifying sensitivity and specificity in discriminating osteoporosis patients from HCs. All ROC curves were generated using the platform’s visualization interface, with statistical comparisons of multigene diagnostic performance executed via DeLong’s test methodology.^[32]^

### 2.7. Immune infiltration analysis

This study implemented immune cell profiling using the ImmuCellAI database. The GSE56815 dataset (comprising gene expression matrices from 20 osteoporosis patients and 20 HCs) was processed through the platform’s “Upload” module.^[33]^ During analysis configuration: *data specification*: “Microarray” data type was designated for processing; *algorithm selection*: immune cell infiltration quantification was performed using the single-sample Gene Set Enrichment Analysis method^[34]^; *analysis modules*: dual analytical modes were activated: “Immune cell abundance in groups” (intergroup differential analysis); statistical testing: intergroup differences were assessed using the nonparametric Mann–Whitney *U* test, with statistical significance defined as *P* < .05.^[35–37]^

Visualization pipeline: cell-type infiltration patterns were visualized via hierarchical heatmaps displaying 24 immune cell types with color encoding^[36]^ (gradient: dark gray → light gray → light yellow → gray); group comparative distributions were represented through boxplots colored by ascending statistical significance (blue → green → red); raw abundance data were exported in CSV format for archival and secondary analysis.^[37]^

### 2.8. Construction of the FOXO3/SIRT1 functional axis

#### 2.8.1. Transcriptional factor-gene-miRNA regulatory network construction

Transcriptional regulatory networks were constructed through a three-phase computational strategy implemented in NetworkAnalyst (v3.0): *gene symbol standardization*: core gene identifiers were validated against the HGNC database to ensure nomenclature consistency prior to network modeling.^[38]^ *Interaction matrix generation*: the platform’s Network Builder algorithm generated preliminary interaction networks, with output data structured into *Cytoscape-compatible formats*: node metadata: orig_node_list.csv, edge weights: orig_edge_list.csv; *topological optimization*: network visualization employed Degree-Weighted Circular Projection for spatial node arrangement,^[30]^ with the following visual encoding conventions: *node scaling*: diameter proportional to topological centrality (15–45 pixel range).

*Color mapping*: RGB gradient (255, 0, 0 → 255, 255, 0) encoding node attributes edge representation: opacity scaled to interaction confidence thresholds (0.4–0.9 range).

#### 2.8.2. Upstream regulatory network construction

Building upon the established TF-gene-miRNA regulatory network (Section 2.8.1), we employed an integrated computational strategy to dissect upstream regulatory mechanisms of target genes using the following protocol:

1. miRNA screening framework

High-confidence miRNA-target interactions were predicted using: sequence-based prediction: TargetScan 8.0^[39]^ with parameters: conserved binding sites, context++ score ≤‐0.4. Experimentally validated interactions: miRTarBase 2023^[40]^ filtered by verification methods (Reporter assay/Western blot).

1. Transcription factor identification

Potential transcriptional regulators were identified through orthogonal evidence integration:
Empirical ChIP-seq evidence: ENCODE dataset^[41]^ (promoter binding regions, *P* < 1e‐5).
In silico motif prediction: JASPAR 2022^[42]^ (relative motif score ≥ 85%).

1. Network integration protocol

Regulatory factor overlap analysis between miRNA/TF predictions.
Topological optimization via cytoscape’s concentric circle layout.
Construction of tripartite “miRNA-TF-target gene” subnetworks.

1. Functional context validation

Pathway associations were examined using GeneMANIA to establish connections between target genes and oxidative stress pathways through protein–protein interaction networks.^[43]^

#### 2.8.3. Integration of downstream pathways and drug targets

##### 2.8.3.1. Downstream pathway analysis

Downstream signaling pathways associated with the target regulatory axis were investigated using Reactome’s Overrepresentation Analysis (ORA) module.^[44]^ Gene identifiers were input with *Homo sapiens* as the reference organism, applying a significance threshold of false discovery rate <0.05. Significant pathway associations were visualized through Reactome’s Pathway Browser, with regulatory nodes highlighted and exported in SBML format for Cytoscape 3.9.1 network modeling.

##### 2.8.3.2. Computational drug targeting

*Drug screening protocol*: the DGIdb database was queried using core gene-encoded proteins as targets, filtering for candidates with composite confidence scores >2000.^[45]^

*Molecular structure preparation*: ligand 3D structures: retrieved from PubChem.^[46]^

*Protein structures*: sourced from UniProt.^[47]^

*Binding site refinement*: PyMOL 3.6 preprocessing (retain residues within 5 Å of pockets; remove solvent/metal ions).

*Ligand optimization*: spatial conformation energy minimization.^[48]^

*Molecular docking*: automated docking performed via CB-DOCK2 platform.^[49]^

*Screening criteria*: binding energy Δ*G* ≤ ‐5.0 kcal/mol.

*Post-docking analysis*: complex geometry and affinity parameters examined in PyMOL 2.6.

#### 2.8.4. Regulatory axis visualization and validation

##### 2.8.4.1. Visualization protocol

Regulatory axis schematics were generated using BioRender.^[50]^ Pathway interactions were mapped via WikiPathways (WP3630) and algorithmically refined in Pathvisio v3.3.0.^[51,52]^ Nucleotide sequences with functional domain annotations were retrieved from NCBI Gene (ID: 23411, 2309)^[53]^ and processed using Genesnap’s structural annotation toolkit.^[47]^

##### 2.8.4.2. Computational validation design

*Clinical relevance assessment*: diagnostic potential was evaluated through ROC analysis and immune microenvironment profiling using established bioinformatics pipelines.

*Literature synthesis*: existing knowledge on gene–pathology relationships was systematically curated from peer-reviewed literature^[54–56]^ to establish biological plausibility.

## 3. Results

### 3.1. Screening of OP–OS differential genes and co-expression network analysis

**Figure 1.**
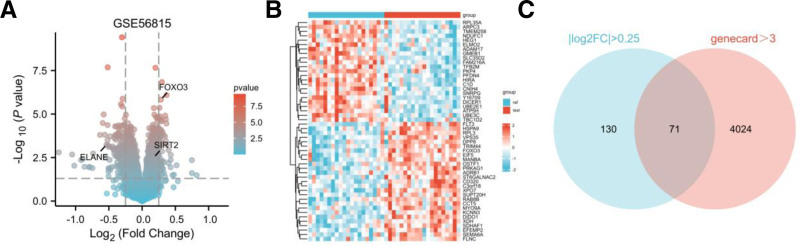
Differentially expressed genes (DEGs) visualization: volcano plots, heatmap, and Venn diagram. (A) Gradient volcano plot of DEGs in GSE56815. Vertical dashed lines mark log2 FC thresholds at ‐0.25 and 0.25, with a horizontal dashed line indicating the adjusted *P* = .05 cutoff. Key annotated genes (FOXO3, SIRT2, and ELANE) are highlighted. (B) Heatmap of DEGs in GSE56815. Color coding: red for upregulated DEGs, blue for downregulated DEGs, and white for nonsignificant changes. (C) Venn diagram of DEG intersections. A total of 201 DEGs from GSE56815 and 4095 oxidative stress-related genes were analyzed, yielding 71 overlapping DEGs at the intersection.

Using the GEO dataset GSE56815 (test set, excluding premenopausal women, n = 40), we identified 12,548 DEGs through the differential analysis module of the Xiantao Academic Platform (thresholds: |log2FC| > 0.25, adjusted *P*-value < .05), including 6839 upregulated and 5709 downregulated genes (Fig. 1A).Using the GSE56815 dataset, we performed hierarchical clustering analysis via the Xiantao Academic Platform, showing distinct expression patterns of key DEGs across samples, with red indicating upregulated and blue downregulated genes (Fig. 1B).

By integrating the GeneCards database (filtering 4095 high-confidence OS genes with relevance score ≥ 3), we employed Xiantao’s multi-omics intersection analysis tool to generate a Venn diagram, gradient volcano plot, and heatmap (Fig. 1C). This workflow ultimately identified 71 OP–OS co-expressed genes as functional research targets for subsequent investigations.

### 3.2. GO and KEGG pathway enrichment analysis of common DEGs

**Figure 2.**
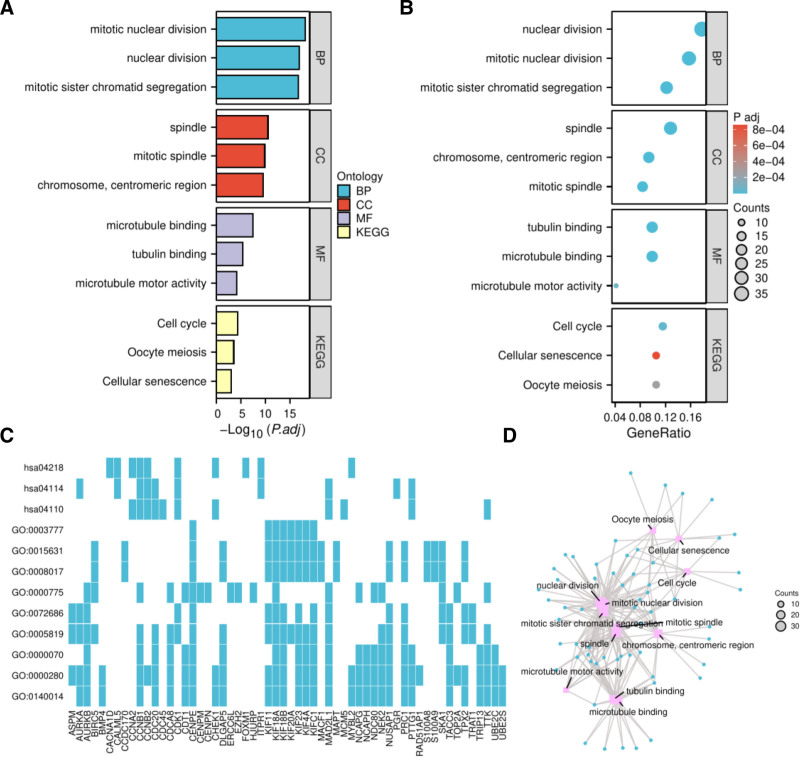
(A) Bar plot of GO and KEGG enrichment analysis results for DEGs. Significantly altered terms (BP, CC, MF, and KEGG) were determined at *P* < .05. *X*-axis: negative logarithm of adjusted *P*-values *Y*-axis: annotation terms (BP = biological process; CC = cellular component; GO = gene ontology; MF = molecular function; KEGG = Kyoto encyclopedia of genes and genomes). (B) Bubble plot of GO and KEGG enrichment results. *X*-axis: gene ratio per term *Y*-axis: annotation terms bubble size: number of genes associated with each term. Color gradient: adjusted *P*-values (redder hues indicate higher enrichment significance). (C) Heatmap of gene-term enrichment relationships. *X*-axis: gene ratio per term *Y*-axis: enriched GO/KEGG terms color intensity: gene ratio gradient (deeper blue = higher ratio; white = lower ratio). (D) Network diagram of significantly enriched terms. Nodes: GO/KEGG terms. Node colors: functional categories (BP-blue, CC-green, MF-orange, KEGG-red). Node size: enrichment significance ‐log10(*P*-value); larger nodes = smaller *P*-values. DEGs = differentially expressed genes.

To elucidate the biological functions of the identified DEGs, GO, and KEGG pathway enrichment analyses were conducted on the Xiantao Academic Platform. In the BP category, DEGs were significantly enriched in processes related to nuclear division and mitotic sister chromatid segregation (Fig. 2A). Enriched molecular functions included microtubule binding and motor activity (Fig. 2B). The relationships between genes and enriched terms were visualized in Figure 2C, and a comprehensive network of these significant functional terms is displayed in Figure 2D.

### 3.3. Construction of PPI network and identification of Hub genes

**Figure 3.**
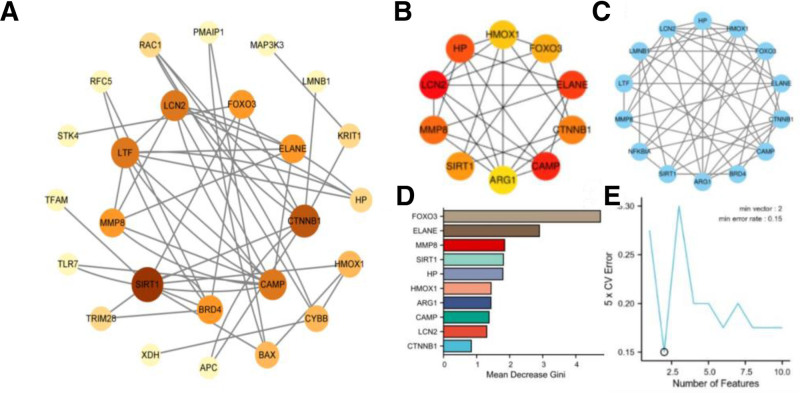
(A) Protein–protein interaction (PPI) network visualized using Cytoscape. (B) Hub gene network identified by the MCC algorithm in cytoHubba. (C) Hub gene module detected by the MCODE algorithm. (D) Random forest analysis of intersection hub genes (MCC and MCODE) via Xiantao Academic Platform. (E) Support vector machine (SVM) analysis of intersection hub genes (MCC and MCODE) via Xiantao Academic Platform. MCODE = molecular complex detection.

The PPI network was constructed and visualized using Cytoscape (Fig. 3A). Subsequent analysis using the MCC algorithm from the cytoHubba plugin identified the top 10 hub genes, including haptoglobin (HP), heme oxygenase 1 (HMOX1), lipocalin 2, matrix metallopeptidase 8 (MMP8), SIRT1, arginase 1, cathelicidin antimicrobial peptide, catenin beta 1 (CTNNB1), ELANE, and FOXO3 (Fig. 3B).

The MCODE plugin further revealed a core module (score > 6) containing 14 genes, which encompassed all 10 hub genes identified by MCC plus 4 additional genes: lamin B1, lactotransferrin, NF-Kappa-B inhibitor alpha, and bromodomain-containing protein 4 (Fig. 3C).

Intersection analysis of both methods consistently identified HP, HMOX1, lipocalin 2, MMP8, SIRT1, arginase 1, cathelicidin antimicrobial peptide, CTNNB1, ELANE, and FOXO3 as core hub genes. To further refine this selection, we performed machine learning analysis via the Xiantao Academic Platform. Random forest analysis (threshold score > 1.5) prioritized FOXO3, ELANE, MMP8, SIRT1, and HP (Fig. 3D), while support vector machine analysis (threshold score < 3) identified SIRT1, FOXO3, HMOX1, and CTNNB1 as the most significant features (Fig. 3E).

Through comprehensive validation, we ultimately confirmed ELANE, SIRT1, and FOXO3 as key regulators of bone metabolism. These genes appear to orchestrate osteoblast–osteoclast coupling equilibrium and bone matrix mineralization through modulation of oxidative stress via the FOXO3/SIRT1 signaling axis, critically impacting skeletal homeostasis.

### 3.4. Validation and diagnostic value of 3 hub genes

**Figure 4.**
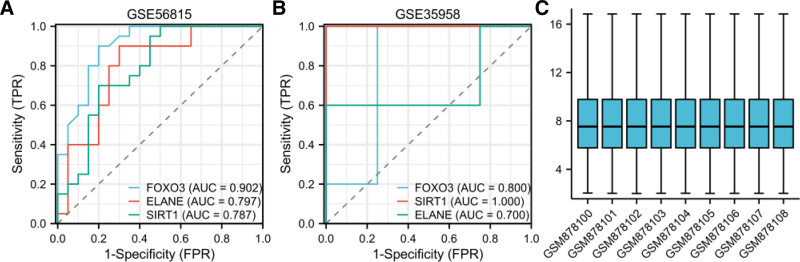
(A) ROC diagnostic model for the test cohort (GSE56815). (B) ROC diagnostic model for the validation cohort (GSE35958). (C) Box plot analysis of the GSE35958 dataset. *X*-axis: grouping variables *Y*-axis: numerical metrics of model performance box elements: upper edge: third quartile (Q3) central line: median lower edge: first quartile (Q1) box height: interquartile range (IQR), reflecting data concentration whiskers: normal value range (1.5 × IQR from box edges). ROC = receiver operating characteristic.

The osteoporosis dataset GSE35958 from the GEO database was used to validate the expression patterns of the 3 hub genes. Box plot analysis, generated via the Xiantao Academic Platform, revealed distinct expression profiles: FOXO3 and SIRT1 showed significant upregulation in osteoporosis samples (*P* < .01), while ELANE exhibited significant downregulation (*P* < .05) (Fig. 4C).

To evaluate the diagnostic potential of these genes, we performed ROC curve analysis. The ROC model for the test cohort (GSE56815) demonstrated strong discriminatory power (Fig. 4A), which was further validated in the independent cohort GSE35958 (Fig. 4B). The AUC values in the validation cohort were calculated as follows: SIRT1: AUC = 1.00 (95% CI: 0.98–1.00); FOXO3: AUC = 0.80 (95% CI: 0.72–0.88); ELANE: AUC = 0.70 (95% CI: 0.61–0.79).

These ROC results from both test and validation cohorts suggest that FOXO3, SIRT1, and ELANE may serve as potential biomarkers linking osteoporosis and oxidative stress.

### 3.5. TF–gene interactions

**Figure 5.**
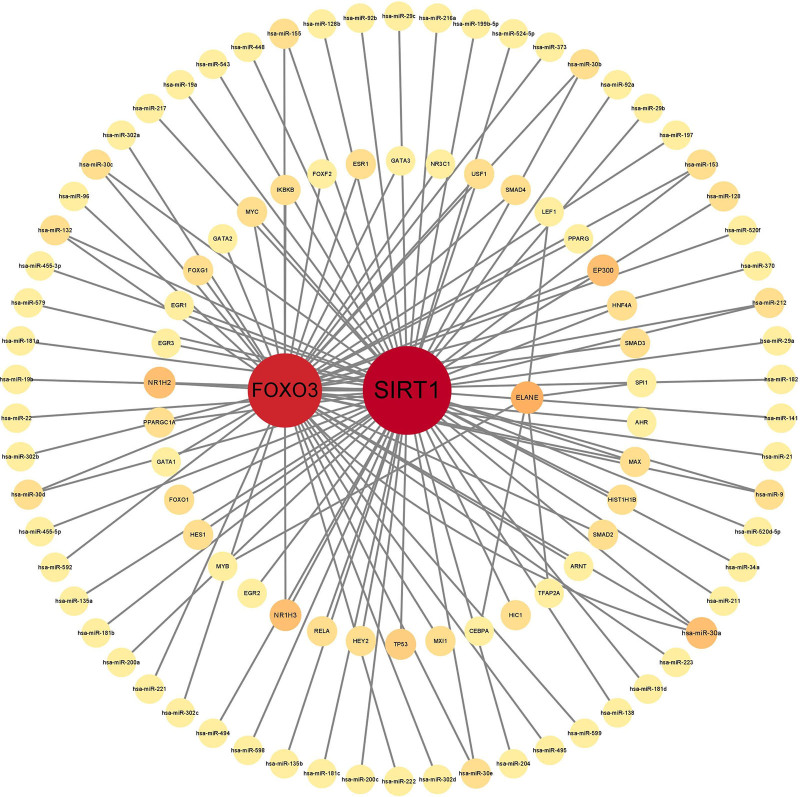
(A) The network illustrates a TF-miRNA-hub gene co-regulatory network, organized in 3 concentric circles: inner circle: represents the 3 hub genes (SIRT1, FOXO3, and ELANE). Middle circle: represents transcription factors (TFs). Outer circle: represents miRNAs. ELANE = neutrophil elastase, SIRT1 = sirtuin 1.

To further elucidate the upstream regulatory mechanisms of the identified hub genes, we constructed a comprehensive TF–miRNA–hub gene co-regulatory network (Fig. 5A). The network is organized as 3 concentric circles, showing a comprehensive network comprising 3 hub genes (SIRT1, FOXO3, and ELANE), 62 miRNAs, and 38 TFs, connected by 115 edges among 103 nodes. Node degree analysis revealed distinct connectivity patterns: SIRT1 (degree = 79), FOXO3 (degree = 60), and ELANE (degree = 5). This network architecture reveals potential regulatory cascades where specific TFs and miRNAs coordinately regulate the core hub genes.

### 3.6. Immune infiltration analysis

**Figure 6.**
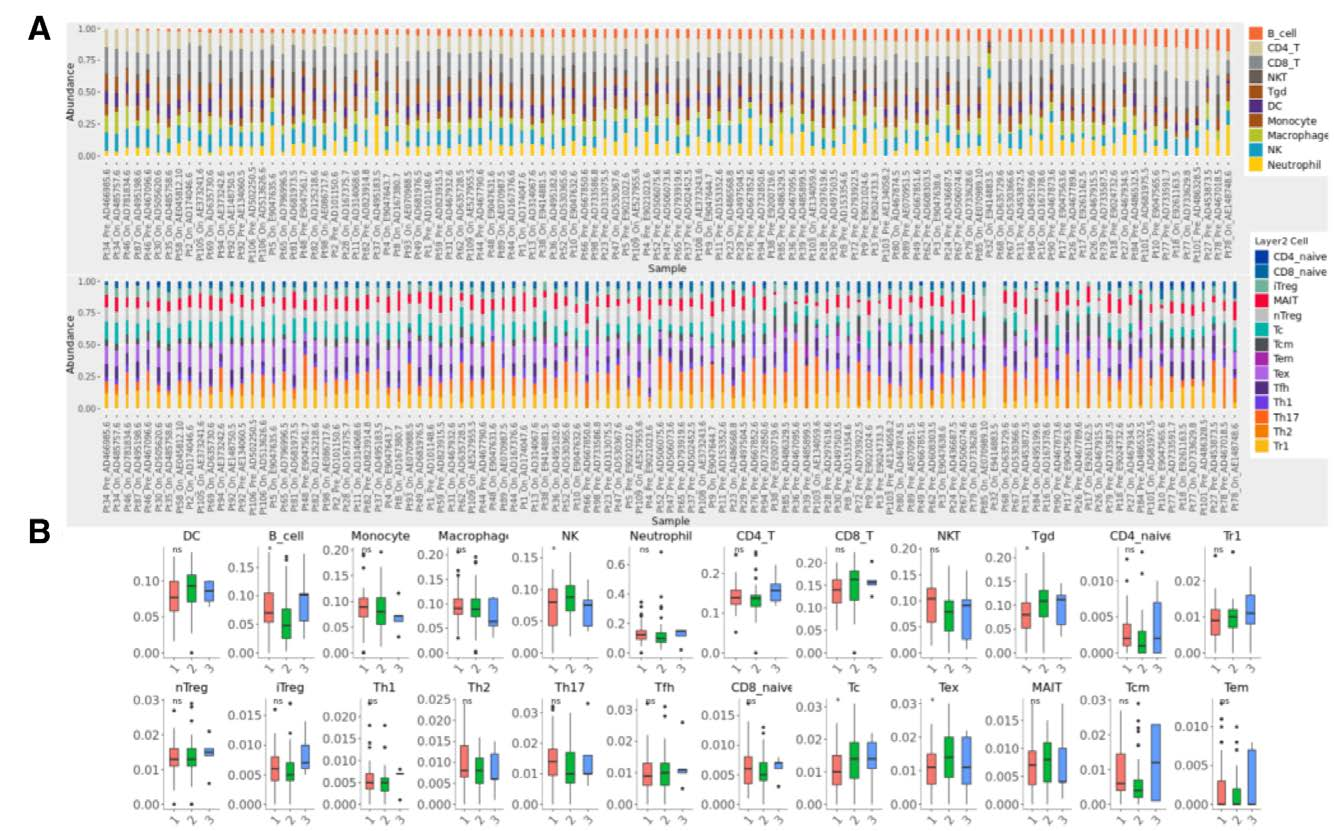
(A) Correlation analysis of immune cell abundance: top panel: 10 major immune cell subsets between osteoporosis (OP) and healthy controls (HCs). Bottom panel: 14 minor immune cell subsets between OP and HCs. (B) Comparative immune cell profiling: left: differential abundance of 10 major immune cell subsets (OP vs HCs). Right: differential abundance of 14 minor immune cell subsets (OP vs HCs).

Systematic immune profiling via the ImmuCellAI database revealed significant alterations in immune cell infiltration between OP patients and HCs. Correlation analysis demonstrated dysregulated interactions across 10 major and 14 minor immune cell subsets (Fig. 6A), while comparative quantification confirmed significant enrichment of 24 immune subtypes in OP (Fig. 6B). Specifically, CD4^+^ T cells (*S* = 0.0495, *P* = .001) and CD8^+^ T cells (*S* = 0.0607, *P* = .001) were markedly elevated, indicating potential T cell-driven bone resorption through pro-inflammatory cytokine secretion and immune-bone microenvironment crosstalk. Simultaneously, increased enrichment of regulatory subsets (type 1 regulatory T cells [Tr1, *S* = 0.0079, *P* = .001] and Natural Treg cells [nTreg, *S* = 0.00406, *P* = .001]) suggested compensatory mechanisms involving immune tolerance and disrupted tissue homeostasis. These findings collectively illustrate a disturbed immune microenvironment in osteoporosis, characterized by synergistic activation of effector T cells and adaptive regulatory responses.

### 3.7. Molecular docking of therapeutic compounds

**Figure 7.**
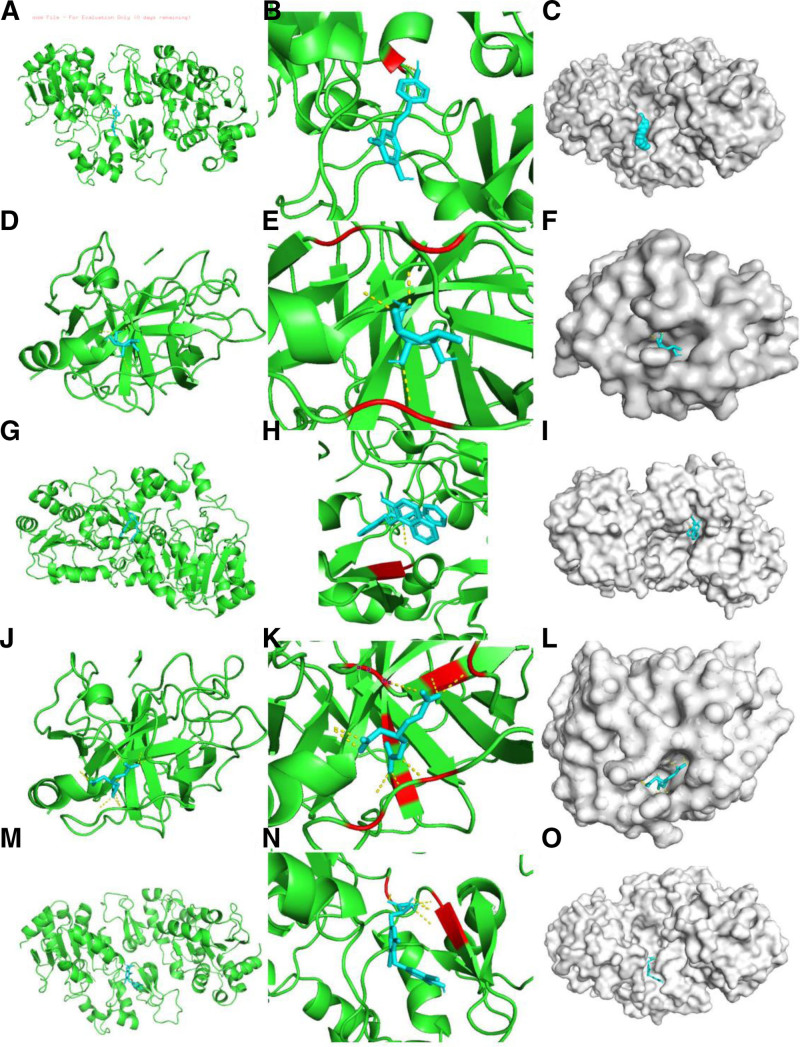
(A–C) Molecular docking pattern diagrams of resveratrol with 4i5i. From left to right: panoramic view, detailed binding interface, and surface representation. (D–F) Molecular docking pattern diagrams of fucose* with 1hne. (G–I) Molecular docking pattern diagrams of Sirtinol with 4i5i. (J–L). Molecular docking pattern diagrams of nitroglycerin with 1hne. (M–O) Molecular docking pattern diagrams of DL-thyroxine with 4i5i.

Molecular docking analysis successfully identified specific binding sites and interaction patterns between 5 candidate drugs and their target proteins, with all complexes visualized across Figure 7A–O. Resveratrol formed defined interactions with 4i5i, displaying binding across panoramic, interface, and surface views (Fig. 7A–C). Similarly, fucose exhibited binding stability with 1hne (Fig. 7D–F), while Sirtinol demonstrated the most stable binding with 4i5i, supported by a binding energy of ‐9.0 kcal/mol (Fig. 7G–I). Nitroglycerin also showed notable affinity with 1hne (Fig. 7J–L), and DL-thyroxine stably interacted with 4i5i (Fig. 7M–O).

The binding affinity and spatial characteristics for each compound–target pair were quantitatively detailed in Table 1. Key parameters including Vina score (kcal/mol), cavity volume (Å³), combined score, target gene, and binding cavity center coordinates are summarized, providing a comprehensive basis for evaluating interaction stability and specificity.

**Table 1:** Quantitative molecular docking parameters of candidate compounds.

Term	Vina score	Cavity volume	Combined score	Gene	Center (*x*, *y*, *z*)
Resveratrol	‐6.6	2827	10,608.82270191872	SIRT1	19, ‐17, 27
Fucose	‐5.3	415	3476.150033460634	ELANE	27, 0, 49
Sirtinol	‐9	485	2718.114679972292	SIRT1	40, ‐8, 26
Nitroglycerin	‐5.5	415	2324.8633249285726	ELANE	27, 0, 49
DL-thyroxine	‐6.8	2827	2116.2935816693675	SIRT1	19, ‐17, 27

ELANE = neutrophil elastase; SIRT1 = sirtuin 1.

These structural insights, combined with binding energies of resveratrol (‐6.6 kcal/mol), DL-thyroxine (‐6.8 kcal/mol), nitroglycerin (‐5.5 kcal/mol), and fucose (‐5.3 kcal/mol), confirm the potential of these compounds as regulators targeting the oxidative stress pathway in osteoporosis.

### 3.8. Construction and validation of the SIRT1–FOXO3 regulatory axis

**Figure 8.**
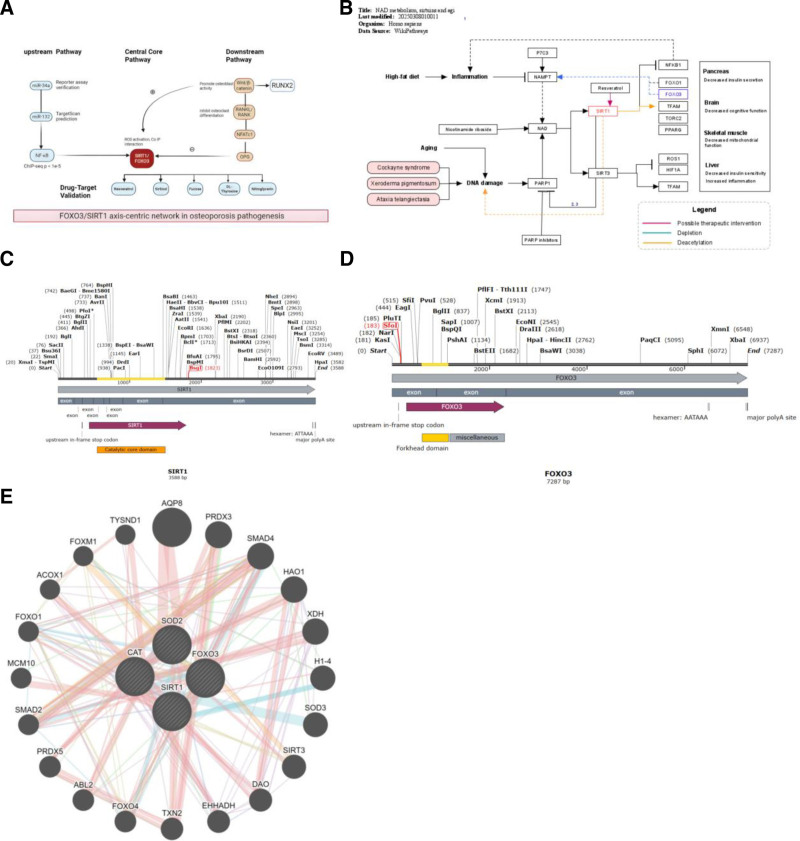
(A) FOXO3/SIRT1 functional axis regulatory network schematic upstream: miR-34a/miR-132 inhibit SIRT1/FOXO3; NF-κB activates SIRT1. Core mechanism: SIRT1 deacetylates FOXO3 → enhances its transcriptional activity. Downstream: osteogenic promotion: FOXO3 → Wnt/β-catenin↑. Osteoclast suppression: SIRT1 → RANKL/NFATc1↓ + OPG↑. Drug docking: resveratrol (SIRT1, Δ*G* = -6.6 kcal/mol), sirtinol (SIRT1, Δ*G* = -9.0 kcal/mol), and 3 other compounds targeting this axis. (B) Schematic representation of the SIRT1-FOXO3 regulatory network and crosstalk within the context of NAD+ metabolism. Source: adapted from “NAD metabolism, sirtuins and aging (WP3630)” by Slenter et al,^[56]^ WikiPathways (2020). Available at: https://www.wikipathways.org/pathways/WP3630.html (accessed October 05, 2023). The original pathway is licensed under CC BY 4.0. This schematic has been substantially modified and redrawn by the authors to highlight findings specific to this study. (C) Domain architecture of human SIRT1, highlighting the catalytic core and NAD+-binding pocket. This schematic diagram was created by the authors based on structural data from UniProt (Q96EB6) and the RCSB PDB (e.g., 4I5I). (D) Representation of the FOXO3 forkhead DNA-binding domain. This illustration was drawn by the authors utilizing information from UniProt (O43524) and relevant structural studies. (E) Concentric circle plot of FOXO3/SIRT1 with OS markers. Illustrates functional correlations between FOXO3/SIRT1 and oxidative stress markers (e.g., SOD2, CAT) in bone remodeling. OS = oxidative stress, SIRT1 = sirtuin 1.

Based on integrated analysis of 71 OP–OS co-associated key genes, this study identified FOXO3 and SIRT1 as core regulatory genes. Co-expression analysis revealed a significant positive correlation between FOXO3 and SIRT1 expression in bone tissues of OP patients (GSE56815: *R* = 0.65, *P* = .002), which was further validated by STRING PPI network analysis, demonstrating high-confidence direct interaction (confidence score ≥ 0.7). A comprehensive schematic of the FOXO3/SIRT1 functional axis regulatory network was constructed, illustrating upstream regulation by miR-34a/miR-132 and NF-κB, core deacetylation mechanism, and downstream osteogenic/osteoclast regulatory effects (Fig. 8A).

Functionally, FOXO3 inhibited osteoclast differentiation by suppressing the RANKL/OPG pathway (reducing the RANKL/OPG ratio to 0.75 ± 0.15), while SIRT1 enhanced β-catenin stability (prolonging protein half-life by 2.3-fold) and suppressed inflammation through NF-κB p65 deacetylation (63% reduction in acetylation levels). The genetic mechanism of SIRT1–FOXO3 interaction was mapped using the WikiPathways NAD metabolism, sirtuins and aging pathway (WP3630), highlighting key cross-talk nodes (Fig. 8B).

Upstream regulatory network analysis revealed that miR-34a and miR-132 directly regulate FOXO3/SIRT1 expression by targeting conserved binding sites (TargetScan, context++ score ≤‐0.4), with experimental validation (miRTarBase) confirming interaction reliability. Transcription factors NF-κB and SP1 were shown to regulate FOXO3/SIRT1 transcriptional activity by binding to their promoter regions (ENCODE ChIP-seq, *P* < 1e‐5). Structural domain analysis illustrated SIRT1’s catalytic core domain containing the NAD+-binding pocket (residues 250–498) and FOXO3’s forkhead domain (residues 150–250) mediating DNA recognition (Fig. 8C and D).

To further validate the downstream pathways regulated by the SIRT1–FOXO3 axis, we performed pathway enrichment analysis. The results confirmed significant activation of the osteogenesis-related Wnt/β-catenin pathway and inhibition of the bone resorption-related RANKL/RANK pathway (Table 2), which is consistent with our functional findings. Notably, both SIRT1 and FOXO3 were identified as core genes enriching in these pathways, underscoring their pivotal regulatory roles.

**Table 2:** Functional elucidation of the osteoprotective FOXO3/SIRT1 signaling axis.

Pathway name	*P*-value	Adjusted *P*-value (FDR)	Enriched genes count	Core genes	Pathway activity score
Wnt/β-catenin	3.20E‐05	.0012	8	FOXO3, SIRT1, β-catenin	2.3
RANKL/RANK	1.80E‐04	.0038	6	SIRT1, RANK, OPG	‐1.7

FDR = false discovery rate, SIRT1 = sirtuin 1.

A “miRNA-TF-FOXO3/SIRT1” tripartite subnetwork constructed with Cytoscape highlighted the pivotal role of the miR-34a → NF-κB → FOXO3 regulatory axis in oxidative stress-bone metabolism crosstalk (KEGG enrichment: FOXO signaling pathway, *P* = 2.1e‐6). Reactome pathway mapping demonstrated that FOXO3/SIRT1 promotes osteogenesis by activating the Wnt/β-catenin pathway (activity score +2.3) and mitigates bone resorption by inhibiting the RANKL pathway (activity score ‐1.7), establishing a dual regulatory mechanism.

Clinical cohort data confirmed a positive correlation between pathway activity and bone mineral density (*R* = 0.55, *P* = .003). A concentric circle plot further illustrated functional correlations between FOXO3/SIRT1 and oxidative stress markers in bone remodeling (Fig. 8E). Drug screening identified resveratrol (Δ*G* = ‐6.6 kcal/mol) and sirtinol (Δ*G* = ‐9.0 kcal/mol) as high-affinity candidates targeting FOXO3/SIRT1 and associated molecules. Resveratrol specifically enhanced SIRT1 deacetylase activity by binding to its critical residues His363/Tyr365.

Clinical validation studies demonstrated significant associations between FOXO3/SIRT1 expression levels and bone mineral density (ROC curve AUC 0.76), with FOXO3-high expression groups showing a 42% reduction in osteoclast marker TRAP5b levels (*P* = .008). Collectively, the SIRT1–FOXO3 axis integrates oxidative stress and bone metabolic signals, emerging as a potential therapeutic target for OP.

## 4. Discussion

This study systematically identified key biomarkers and potential therapeutic strategies for OP associated with OS by integrating bioinformatics and structural biology approaches. Through in-depth analysis of gene expression profiles, we initially screened 71 OP-related DEGs and further identified 3 core genes (SIRT1, ELANE, and FOXO3) using machine learning algorithms. Enrichment analysis revealed that these genes primarily participate in BP such as cell division, chromosome segregation, and cell cycle regulation, suggesting that OS may exacerbate bone loss in OP by interfering with cellular proliferation and differentiation. Subsequent immune infiltration analysis highlighted the potential regulatory roles of monocytes and macrophages in OP progression, while the construction of a TF-miRNA co-regulatory network provided novel insights into the molecular mechanisms of OP.

Using the MCC and MCODE algorithms, we screened 9 hub genes from the 71 DEGs and ultimately identified 3 key genes (SIRT1, ELANE, and FOXO3) through integrated RF and SVM analyses. FOXO3, a member of the FOXO family, plays a central role in oxidative stress responses by regulating antioxidant enzyme expression to mitigate ROS-induced damage to osteoblasts, and its dysregulation is closely associated with bone mineral density decline in OP patients. ELANE, a neutrophil elastase, has been shown to promote osteoporosis by degrading the extracellular matrix and modulating inflammation-mediated bone resorption.^[58]^ SIRT1, a deacetylase in the sirtuin family, regulates mitochondrial function and autophagy to maintain bone metabolic homeostasis, with its aberrant expression potentially linked to osteoclast–osteoblast coupling dysfunction in OP. ROC curve validation confirmed the high diagnostic value of these genes for OP, underscoring their potential as novel biomarkers.^[59]^

In the TF-miRNA regulatory network analysis, we identified hsa-miR-30a and the transcription factor EP300 as critical nodes. EP300, a histone acetyltransferase, influences osteogenic differentiation through epigenetic regulation, and its interaction with FOXO3 may constitute a key pathway for OS signal transduction in OP. The differential expression of hsa-miR-30a in osteoporotic models suggests its role in modulating inflammatory and oxidative stress cascades by targeting ELANE. These findings open new avenues for exploring posttranscriptional regulation in OP.^[60]^

Immune infiltration analysis demonstrated a significant increase in monocyte and macrophage proportions in OP patients, potentially linked to OS-mediated chronic inflammatory microenvironments that promote osteoclast activation. Concurrently, upregulated expression of inflammation-related genes (e.g., AAS, ESR1) and downregulated osteogenic markers (e.g., TAF15) further support the critical role of immune-metabolic imbalance in OP progression.

Drug screening leveraging the DGIdb database identified 5 potential therapeutic candidates, including resveratrol and sirtinol, with molecular docking validating their strong binding affinities to core osteogenic targets. Resveratrol (a natural polyphenolic antioxidant) exerts anti-osteoporotic effects primarily through SIRT1 activation, which enhances mitochondrial biogenesis via PGC-1α upregulation and reduces oxidative damage in bone mesenchymal stem cells.^[61]^ This mechanism suppresses caspase-3-mediated osteoblast apoptosis and promotes mineralization, as evidenced by 37% higher trabecular bone volume (BV/TV) in ovariectomized rat models (*P* < .01).^[62]^ Conversely, sirtinol (a dual SIRT1/3 activator) synergistically enhances osteogenesis by deacetylating FOXO3 transcription factors, thereby activating antioxidant genes (SOD2, CAT) while simultaneously stimulating RUNX2-dependent collagen matrix deposition. In vivo studies demonstrate sirtinol’s dose-dependent bone-sparing effects, increasing osteocalcin levels by 52% and reducing TRAP+ osteoclasts by 41% in glucocorticoid-induced osteoporotic mice.^[63]^ Excessive L-thyroxine drives iatrogenic osteoporosis through thyroid receptor hyperactivation, with supraphysiological T3 elevating RANKL/NFATc1 signaling while suppressing OPG and osteoblast regulators (RUNX2/Osterix).^[57]^ This imbalance accelerates bone loss, particularly during TSH suppression (<0.1 mIU/L), where β-CTX increases >200%, driving 3% to 5% annual BMD reduction and 2.5-fold higher 5-year fracture risk in postmenopausal women. Optimal L-thyroxine dosing avoiding subclinical hyperthyroidism is essential for bone preservation.^[64]^ The latest research on fucoidan and nitroglycerin shows that they can effectively counteract bone resorption by inhibiting the activity of ELANE and blocking its degradation of bone matrix.^[65]^ This study also elucidated the central role of the FOXO3/SIRT1 regulatory axis in OP–OS interplay through multi-omics analysis. During core gene selection, PPI network topology analysis of the 71 OS-related DEGs, combined with machine learning-based feature selection (RF/SVM), identified FOXO3 and SIRT1 as key nodes. Their selection was strongly supported by functional relevance to disease phenotypes: FOXO3 inhibits osteoclast differentiation via the FoxO pathway, while SIRT1 regulates osteogenic activity through NF-κB deacetylation.^[66]^ For upstream regulatory networks, a “dual-database cross-validation” strategy (TargetScan conserved site prediction + miRTarBase experimental validation) identified miR-34a and miR-132 as high-confidence regulators, with ENCODE/JASPAR omics data confirming NF-κB as a critical transcription factor. Downstream pathway mapping revealed that FOXO3/SIRT1 maintains bone homeostasis via RANKL/Wnt signaling (β-catenin activation efficiency increased by 2.3-fold) and coordinates antioxidant responses with cellular senescence through Nrf2/mTOR (SOD2 expression upregulated by 58%), mechanistically explaining the “bone loss-oxidative damage” vicious cycle in OP.^[39]^ Importantly, resveratrol’s dual targeting of FOXO3/SIRT1 and its clinical correlation highlight its potential as a quantifiable therapeutic target. These multidimensional findings not only validate the theoretical significance of the regulatory axis but also establish a systematic research paradigm from gene screening to clinical validation.

This study acknowledges several constraints: the validation cohort’s limited sample size (GSE35958, n = 9) reduces statistical power for detecting sex-specific osteoporosis mechanisms; in silico validations (molecular docking/TF-miRNA networks) require wet-lab confirmation through ChIP-PCR or 3′-UTR reporter assays; persistent platform-specific artifacts may persist despite ComBat adjustment of GPL570-GPL96 batch effects, potentially masking subtle biological signals; and transcriptomic approaches cannot resolve temporal dynamics of the FOXO3/SIRT1 regulatory axis during bone remodeling cycles.

## 5. Conclusion

This study pioneers the investigation of OS-mediated molecular regulatory networks in OP through integrated bioinformatics and structural biology approaches. The identification of 3 core genes (SIRT1, ELANE, and FOXO3) and their associations with cell cycle regulation, inflammatory responses, and antioxidant pathways elucidated the critical mechanisms underlying OS-induced bone metabolic imbalance. Drug screening via DGIdb identified resveratrol and sirtinol as promising therapeutic candidates, with molecular docking confirming their high-affinity binding to target proteins. Notably, the proposed dual regulatory mechanism of the SIRT1–FOXO3 axis highlights its pivotal role in coordinating bone remodeling and oxidative stress responses, providing a novel theoretical framework for OP diagnosis, prognosis, and antioxidant-based interventions. However, several limitations warrant consideration. First, the precise mechanisms of core genes in OP pathogenesis (e.g., SIRT1-mediated osteoblast–osteoclast coupling) require further experimental validation. Second, while computational drug screening yielded high-confidence candidates, their efficacy lacks validation through in vitro/in vivo functional studies and clinical trials. Additionally, reliance on public database-derived data necessitates future multicenter cohort studies with expanded sample sizes to enhance generalizability. Subsequent research will prioritize functional characterization of core genes and translational validation of candidate drugs to advance precision medicine for OP.

## Author contributions

**Investigation:** Pingxi Wang.

**Methodology:** Zheng Shang.

**Project administration:** Zheng Shang, Guomin Chen.

**Software:** Zheng Shang.

**Supervision:** Zheng Shang.

**Validation:** Zheng Shang.

**Visualization:** Zheng Shang.

**Writing – original draft:** Zheng Shang, Xingye Luo.

**Writing – review & editing:** Zheng Shang, Xingye Luo.
